# Metabolic potential of the imperfect denitrifier *Candidatus* Desulfobacillus denitrificans in an anammox bioreactor

**DOI:** 10.1002/mbo3.1227

**Published:** 2021-08-28

**Authors:** Takashi Okubo, Hideto Takami

**Affiliations:** ^1^ Marine Microbiology, Atmosphere and Ocean Research Institute The University of Tokyo Kashiwa Japan

**Keywords:** anammox bioreactor, *Ca*. Desulfobacillus denitrificans, denitrification, metabolic pathway

## Abstract

The imperfect denitrifier, *Candidatus* (*Ca*.) Desulfobacillus denitrificans, which lacks nitric oxide (NO) reductase, frequently appears in anammox bioreactors depending on the operating conditions. We used genomic and metatranscriptomic analyses to evaluate the metabolic potential of *Ca*. D. denitrificans and deduce its functional relationships to anammox bacteria (i.e., *Ca*. Brocadia pituitae). Although *Ca*. D. denitrificans is hypothesized to supply NO to *Ca*. B. pituitae as a byproduct of imperfect denitrification, this microbe also possesses hydroxylamine oxidoreductase, which catalyzes the oxidation of hydroxylamine to NO and potentially the reverse reaction. *Ca*. D. denitrificans can use a range of electron donors for denitrification, including aromatic compounds, glucose, sulfur compounds, and hydrogen, but metatranscriptomic analysis suggested that the major electron donors are aromatic compounds, which inhibit anammox activity. The interrelationship between *Ca*. D. denitirificans and *Ca*. B. pituitae via the metabolism of aromatic compounds may govern the population balance of both species. *Ca*. D. denitrificans also has the potential to fix CO_2_ via an irregular Calvin cycle and couple denitrification to the oxidation of hydrogen and sulfur compounds under chemolithoautotrophic conditions. This metabolic versatility, which suggests a mixotrophic lifestyle, would facilitate the growth of *Ca*. D. denitrificans in the anammox bioreactor.

## Graphical abstract

The imperfect denitrifier *Ca*. D. denitrificans inhabiting anammox bioreactors can use a range of electron donors for denitrification, including aromatic compounds, glucose, sulfur compounds, and hydrogen. However, our metatranscriptomic analysis suggested that the major electron donors are aromatic compounds, which inhibit anammox activity.

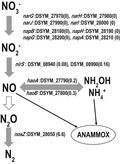

## 1 INTRODUCTION

In a previous study, we successfully reconstructed the whole‐genome sequences of three currently non‐isolatable major community members in an anaerobic ammonium oxidation (anammox) bioreactor, *Candidatus* (*Ca*.) Brocadia pituitae, *Ca*. Nitrosymbiomonas proteolyticus, and *Ca*. Desulfobacillus denitrificans (Okubo et al., 2021). Through comparative genomics of anammox bacteria, it was found that *Ca*. Brocadia pituitae lacked any genes encoding a canonical nitrite reductase (i.e., *nirK* and *nirS*), but possessed candidate genes (*hao2* and *hao3*) encoding NO‐forming or NH_2_OH‐forming nitrite reductases. Also, analysis of the partial genome sequence of *Ca*. D. denitrificans revealed that this microbe is likely to be a nitric oxide (NO)‐forming incomplete denitrifier because NO reductase genes (*norBC*) are missing, although a *nirS* gene was detected in the genome. Thus, it was hypothesized that *Ca*. B. pituitae uses not only self‐produced NO and/or NH_2_OH, but also NO supplied by incomplete denitrifiers such as *Ca*. D. denitrificans. *Ca*. D. denitrificans has frequently been detected in other anammox bioreactors (Bae et al., 2010; Lawson et al., 2017) and its genome showed 99.0%‐average nucleotide identity (ANI) to the draft genome of *Rhodocyclaceae* bacterium UTPRO2 from the metagenome of an anammox bioreactor (Lawson et al., 2017).

Recently, it has been reported that denitrification synergized with anammox could accelerate the anaerobic degradation of benzene, and *Rhodocyclaceae* bacteria might play a role in benzene degradation (Peng et al., 2017). Although one possible contribution of anammox bacteria could be to remove the nitrite that accumulates as a result of denitrification, benzene and metabolic intermediates such as toluene, phenol and benzoate were found to inhibit anammox activity (Peng et al., 2018). In addition to toxic aromatic compounds, anammox activity is also inhibited by non‐toxic organic matter, salts, heavy metals, phosphate, and sulfide, which are commonly present in practical applications such as wastewater treatment (Jin et al., 2012). On the other hand, because 16S rRNA genes with more than 99% identity to that of *Ca*. N. proteolyticus have also been detected in many other anammox bioreactors (Liu et al., 2009; Park et al., 2017), this aerobic species was inevitably suggested to be an anammox bacterial community (ABC) member responsible for nitrite oxidation via consumption of O_2_ in the anammox bioreactor (Okubo et al., 2021). Since *Ca*. N. proteolyticus possesses multiple secretory lytic enzymes and type II secretion systems, it was suggested that proteolysis of biomass from autolyzed cells and also the lysis of active cells sensitive to lytic enzymes may provide nutrients for itself as well as other heterotrophic members of the ABC (Okubo et al., 2021). Therefore, these predominant bacteria may be important cooperators that help to maintain a balanced population of ABC members and stable anammox activity in the bioreactor. Indeed, cooperative relationships were suggested in cross‐feedings of nutrients such as amino acids, carbohydrates, and vitamins, and also in cell aggregation by supplying exopolysaccharides (Lawson et al., 2017; Zhao et al., 2019). However, the lifestyles of major cooperators in the ABC are still unclear, as complete genome sequences of non‐isolatable cooperators have not yet been obtained although NO production by incomplete denitrification is not unusual (Schuster & Conrad, 1992). In this study, we performed a detailed genomic analysis of *Ca*. D. denitrificans and examined the expression profile of its genes to determine why *Ca*. D. denitrificans is selected as a predominant species in the ABC and how it interacts with anammox bacteria, that is, *Ca*. B. pituitae.

## 2 MATERIALS AND METHODS

### 2.1 Genomic sequence

The ABC comprised members of four major species, anammox bacteria, *Ca*. B. pituitae (35%), nitrite‐oxidizing bacteria, *Ca*. N. proteolyticus (10%) and imperfect denitrifiers, *Ca*. D. denitirificans (7%), and *Ca*. Denitrolinea symbiosum (3.4%) (Okubo et al., 2021). Among them, the genome sequence of *Ca*. D. denitrificans was used for detailed analysis in this study. To determine the phylogenetic position of *Ca*. D. denitrificans, a total of forty complete or draft genome sequences of bacteria, classified mainly into the orders *Rhodocyclales*, *Burkholderiales*, and *Nitrosomonadales*, were obtained from the DDBJ/EMBL/GenBank database.

### 2.2 Evaluation of the metabolic and physiological potential

The pattern of the metabolic and physiological potential of *Ca*. D. denitrificans was investigated using Genomaple^TM^ (formerly MAPLE) ver. 3.2 (Arai et al., 2018; Takami et al., 2016). Genomaple^TM^ is available through a web interface (https://maple.jamstec.go.jp/maple/maple‐2.3.1/) and as a stand‐alone package from Docker Hub (https://hub.docker.com/r/genomaple/genomaple). Genes were mapped to 795 functional modules defined by the KEGG (pathways, 305; complexes, 294; functional sets, 157; and signatures, 40), and the module completion ratio (MCR) was calculated according to a previously described Boolean algebra–like equation (Takami et al., 2012). To evaluate the MCR, *Q*‐values suggesting the working probability of the modules were also calculated by Genomaple^TM^. *Q*‐values near zero indicate a high working probability of the module (Takami et al., 2016).

### 2.3 Analysis of RNA‐seq data

To identify the actively working metabolic pathways in the anammox reactor, metatranscriptomic reads obtained in a previous study (Okubo et al., 2021) were mapped to the genome sequences with a cutoff identity of 95% using the Magic‐BLAST program (Boratyn et al., 2019). The numbers of mapped reads were counted by SAMtools (Li et al., 2009) and HTSeq (Anders et al., 2015). The RPKM (reads per kilobase of exon per million mapped sequence reads) ratio, calculated by dividing the RPKM of each gene by the mean RPKM of all ribosomal proteins, was used to determine relative gene expression levels. Physiological and biochemical features of hydrogenases were estimated with the HydDB program (Søndergaard et al., 2016).

### 2.4 Phylogenetic analysis

Amino acid sequences of 45 ribosomal proteins, commonly detected by Genomaple^TM^ ver. 2.3.0 (Arai et al., 2018; Takami et al., 2016) except for RpsN (K02954), RpmJ (K02919), RpsD (K2986), RpmH (K01914), RplI (K02939), RpsP (K02959), and RplS (K02884), were concatenated and aligned by the MUSCLE program (Edgar, 2004). A phylogenic tree based on concatenated aligned sequences was constructed to determine the phylogenetic position of *Ca*. D. denitrificans by the maximum likelihood (ML) method with the LG+G+I+F model in the MEGA 10.1.8 package (Kuma et al., 2018). *Vulcaniibacterium thermophilum* KCTC 32020 (GCA_007923255.1) was used as an outgroup. A phylogenetic tree based on hydroxylamine oxidoreductase (Hao)‐like proteins was constructed by the ML method with the WAG + G + I model with 500 bootstrap replicates. The oxidative and reductive types of dissimilatory sulfite reductase (*dsrAB*) were classified by the neighbor‐joining method in the same package. The concatenated DsrAB amino acid sequence dataset (Müller et al., 2015) was used as a reference to infer the position of DsrAB from *Ca*. D. denitrificans.

## 3 RESULTS AND DISCUSSION

### 3.1 Denitrification pathway

**FIGURE 1:**
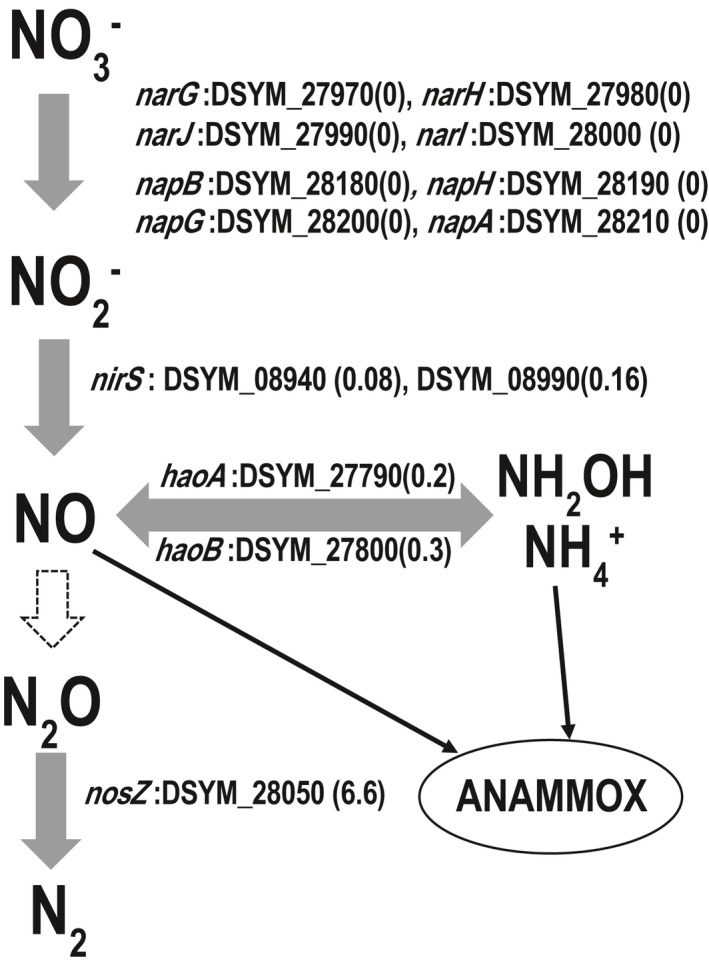
Nitrogen metabolism pathway. Values in parentheses represent RPKM ratios. The dotted arrow shows the missing process. By‐products though imperfect denitrification such as NO, NH_2_OH, and NH_4_^+^ are thought to be supplied to anammox bacteria

*Ca*. D. denitrificans possesses all genes necessary for denitrification except for NO reductase (i.e., *norBC*) as shown in Figure 1. Also, *narXL* (DSYM_27930‐27940), which are presumably involved in nitrate/nitrite dependent transcriptional activation of genes encoding nitrate oxidoreductase (i.e., *narGHJI*; DSYM_27970‐8000) (Härtig et al., 1999) and the formate/nitrite transporter gene (DSYM_27950) were located upstream of *narGHJI* (Table S1: https://doi.org/10.5281/zenodo.5089211). Among these genes for nitrogen metabolism, expression of the genes encoding nitrite reductase (*nirS*) and nitrous oxide reductase (*nosZ*) was observed under the regular operating conditions of the anammox bioreactor (Figure 1 and Table S1: https://doi.org/10.5281/zenodo.5089211). The expression level of *nosZ* was much higher than that of *nirS*. Accordingly, it is thought that nitrous oxide (N_2_O) is used as a major electron acceptor in *Ca*. D. denitrificans; however, unexpectedly *Ca*. D. denitrificans lacks *norBC*, which encodes the enzyme responsible for the reduction of NO to N_2_O. Thus, we explored the possibility that N_2_O is produced by alternative enzymes. It is known that nitric oxide reductase is structurally similar to cytochrome oxidase (Zumft, 1997) and indeed, the cytochrome *cbb_3_*‐type oxidase of P*seudomonas stutzeri* is known to have nitric oxide reductase activity (Forte et al., 2001). Although *Ca*. D. denitrificans possesses two genes encoding a cytochrome *cbb_3_*‐type oxidase (Table S2: https://doi.org/10.5281/zenodo.5089211), expression of these genes was not detected. On the other hand, NO‐detoxifying enzymes can also reduce NO to N_2_O (Gardner et al., 2003), but *Ca*. D. denitrificans has no genes encoding this enzyme (*norVW*). These results suggest that *Ca*. D. denitrificans does not utilize self‐produced N_2_O, but presumably it can use N_2_O supplied by other community members because *norBC* genes from minor community members were detected in the anammox bioreactor (Okubo et al., 2021) although alternative ways of producing N_2_O have been reported in other heterotrophic nitrifying bacteria (Zhang et al., 2015), aerobic ammonium oxidizing bacteria (AOB) (Caranto et al., 2016). On the other hand, since the *nosZ* gene is highly expressed, *Ca*. D. denitrificans is thought to reduce N_2_O emissions from the anammox bioreactor. It has been reported that only 0.0037% of the total nitrogen load in the anammox reactor was emitted as N_2_O even though N_2_O was detected within anammox granules (Rathnayake et al., 2018). N_2_O has a greenhouse effect more than 300 times that of carbon dioxide on a 100‐y timescale and it also depletes the ozone layer (Solomon et al., 2007).

**FIGURE A1:**
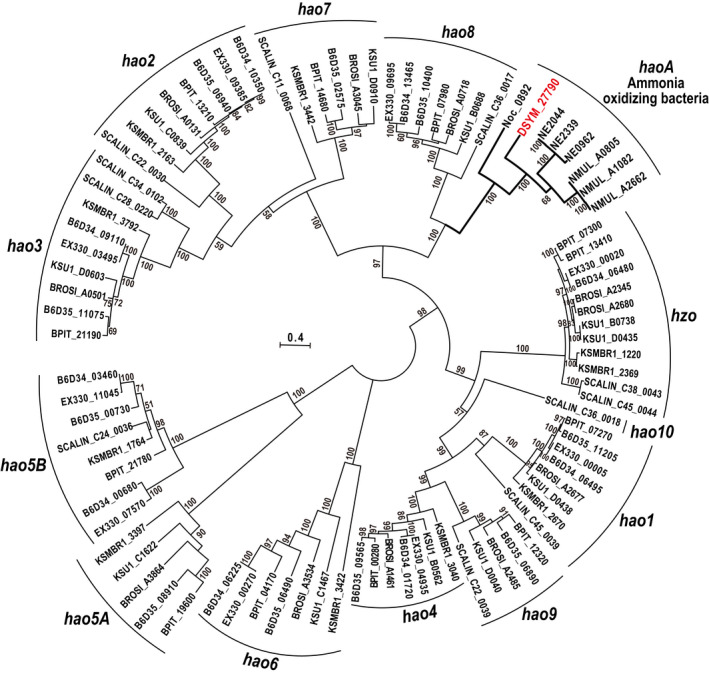
Phylogenetic tree based on hydroxylamine oxidoreductase (Hao)‐like proteins. Hao proteins from aerobic and anaerobic ammonia‐oxidizing bacteria were used in this figure as the reference proteins. Noc, *Nitrosococcus oceani* ATCC19707; NE, *Nitrosomonas europaea* ATCC 19718; Nmul, *Nitrosospira multiformis* ATCC 25196; BPIT, *Ca*. Brocadia pituitae; BROSI, *Ca*. B. sinica JPN1; EX330, *Ca*. Brocadia sp. BROELEC01; B6D34, *Ca*. Brocadia sp. UTAMX1; B6D35, *Ca*. Brocadia sp. UTAMX2; KSU1, *Ca*. Jettenia caeni KSU1; KSMBR1, *Ca*. Kuenenia stuttgartiensis MBR1; SCALIN, *Ca*. Scalindua japonica husup‐a2. The red character shows Hao protein from *Ca*. Desulfobacillus denitrificans

The *haoA* (DSYM_27790) gene product (i.e., hydroxylamine oxidoreductase) from *Ca*. D. denitrificans clustered with those of aerobic AOB in the genera *Nitrosospira* and *Nitrosomonas* (Figure A1). The *haoB* (DSYM_27800) gene, encoding another whose functional role remains to be elucidated, is adjacent to *haoA* and the genes encoding cytochrome *c*_554_ (DSYM_27810) and cytochrome *c* (DSYM_27820), proteins that would relay the electrons to the quinone pool. Among these neighboring genes, only *haoA* and *haoB* were expressed (Figure 1). Although the Hao protein catalyzes the oxidation of hydroxylamine to NO (Caranto & Lancaster, 2017) in aerobic AOB, it has also been reported to catalyze the reduction of NO to hydroxylamine and ammonium, which are substrates for anammox bacteria (Kostera et al., 2010). Accordingly, *Ca*. D. denitrificans is thought to provide not only NO, but also hydroxylamine and ammonium to anammox bacteria. However, because the reaction pathway catalyzed by the Hao protein is still unclear, further study on the role of this protein in *Ca*. D. denitrificans is required to fully understand the nitrogen flow in the anammox community.

### 3.2 Aromatic compound metabolism

**FIGURE 2:**
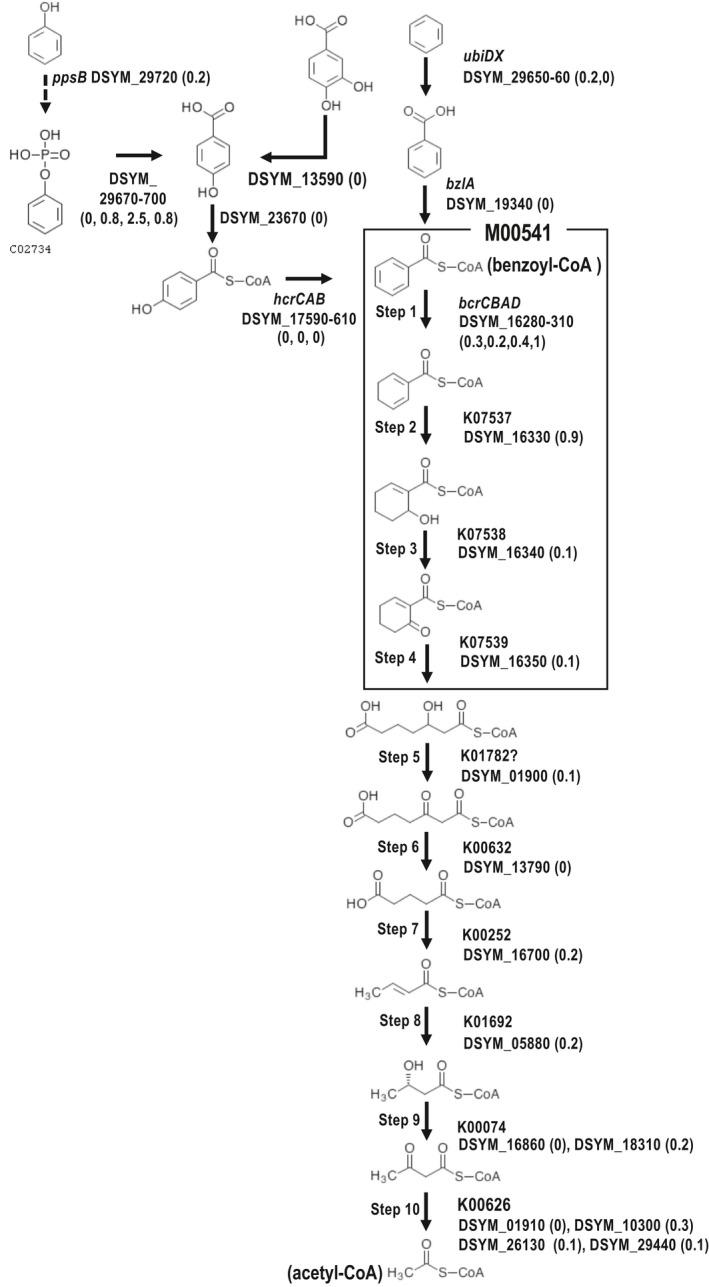
Anaerobic pathway for the degradation of aromatic compounds. The gene encoding the phenylphosphate synthase alpha subunit (PpsA) was missing. M00541: module for Benzoyl‐CoA degradation (benzoyl‐CoA => 3‐hydroxypimeloyl‐CoA). The dotted line shows the reaction by the incomplete phenylphosphate synthase (*pss*) lacking subunit A

**FIGURE 3:**
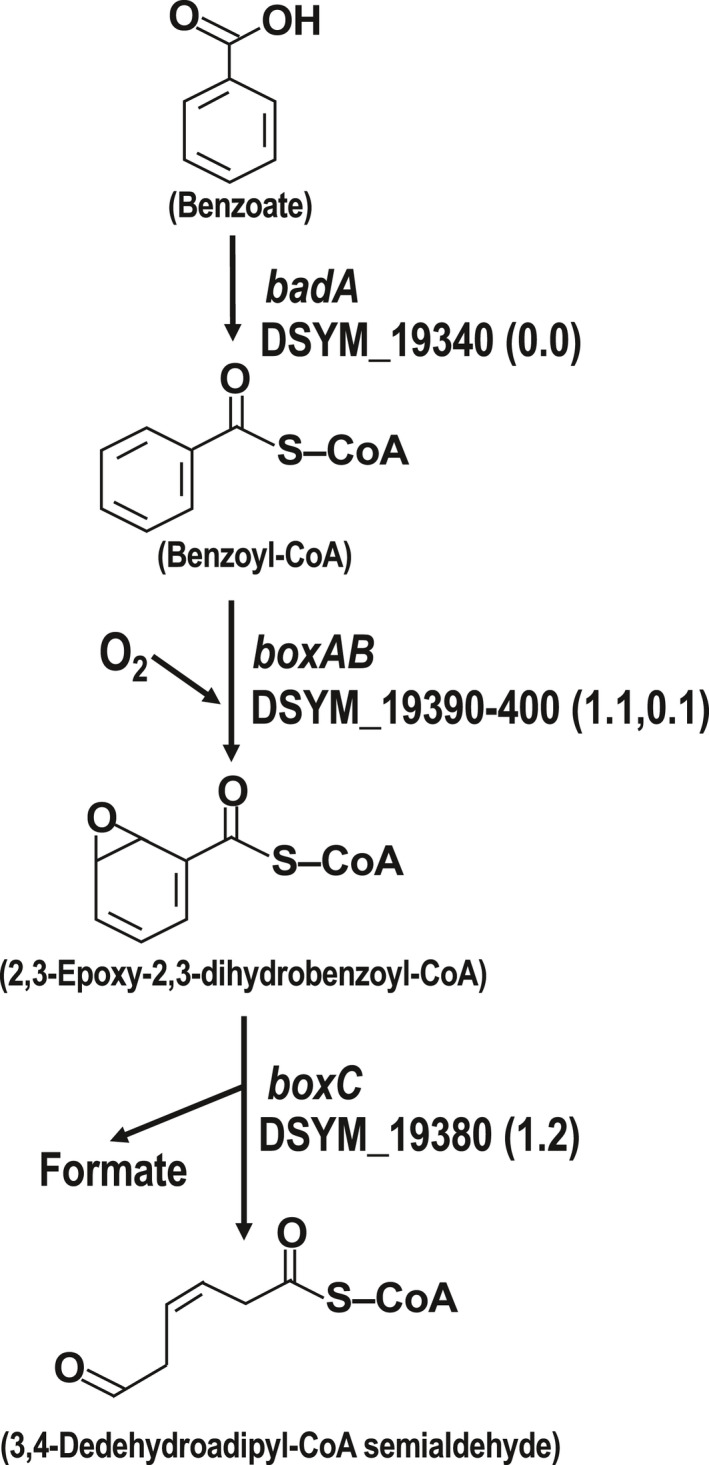
Aerobic pathway for benzoate degradation. Values in parentheses represent RPKM ratios

**FIGURE A2:**
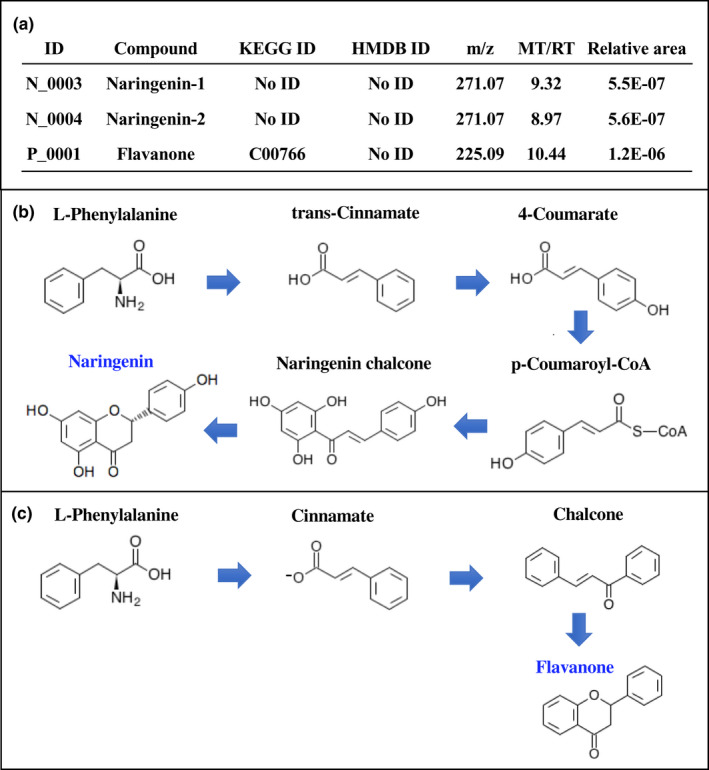
Aromatic compound detected in the centrifugal supernatant of wet biomass. (a) Detection of aromatic compounds by LC‐TOFMS. P (positive mode), N (negative mode), Measurement conditions are as follows. LC system: Agilent 1200 series RRLC system SL, Column: ODS column, 2 × 50 mm, 2 μm, MS system: Agilent LC/MSD TOF, Column temp.: 40℃, Mobile phase (a: H_2_O/0.1% HCOOH, b: Isopropanol: Acetonitrile: H_2_O (65:30:5)/0.1% HCOOH, 2 mM HCOONH_4_), Flow rate: 0.3 mL/min, Run time: 20 min, Post time: 7.5 min, Gradient condition: 0–0.5 min: B 1%, 0.5–13.5 min: B 1–100%, 13.5–20 min: B 100%, MS ionization mode: ESI negative or positive, MS Nebulizer pressure: 40 psi, MS dry gas flow: 10 L/min, MS dry gas temp: 350℃, MS capillary voltage: 3500 V, MS scan range: m/z 100–1,700, Sample injection: 1 μL. m/z, molecular mass/charge; MT, migration time; RT, retention time; KEGG, Kyoto Encyclopedia of Genes and Genomes; HMDB, Human Metabolome Database. (b) Synthetic pathway for naringenin. (c) Synthetic pathway for flavanone

A wide range of organic and inorganic compounds can act as electron donors for denitrifying bacteria (Capua et al., 2019), although aromatic compounds are known to inhibit anammox activity and decrease the population of anammox bacteria (Peng et al., 2018; Pereira et al., 2014). Here, we predicted the preferred electron donors for the incomplete denitrifier, *Ca*. D. denitrificans under the regular operating conditions of the anammox bioreactor (Okubo et al., 2021) based on genomic and transcriptomic data. *Ca*. D. denitrificans contains a complete module for the ATP‐dependent benzoyl‐CoA degradation pathway (M00541), which converts benzoyl‐CoA to 3‐hydroxypimeloyl‐CoA (Figure 2 and Table 1). Benzoyl‐CoA is a key in the anaerobic degradation of many aromatic compounds (Fuchs et al., 2011). Expression of the genes encoding the conversion of 3‐hydroxypimeloyl‐CoA to acetyl‐CoA (step 5–10 in Figure 2) was also observed except for step 6. The acetyl‐CoA generated through this pathway would be used in the TCA cycle and glyoxylate cycle for not only ATP and NADH production but also carbon assimilation (Table 1). Transcripts for the gene encoding step 6 may have been undetected because the number of transcript reads that mapped to genes associated with this module was small overall. *Ca*. D. denitrificans also possessed all of the genes necessary for the metabolism of benzene, benzoate, phenylphosphate, 4‐hydroxybenzoate, and protocatechuate into benzoyl‐CoA (Figure 2). *Ca*. D. denitrificans is thought to be a member of a new family within the order *Rhodocyclales*. The genes encoding formate dehydrogenase‐N (see Phylogenetic position section), but other bacteria within this order have also been detected in benzene‐degrading nitrate‐reducing microbial consortia (Atashgahi et al., 2018; Zaan et al., 2012). *Ca*. D. denitrificans also possessed genes encoding benzoate‐CoA ligase (*badA*) and benzoyl‐CoA 2,3‐epoxidase (*boxABC*), which catalyze the degradation of benzoate to 3,4‐dehydroadipyl‐CoA semialdehyde and formate under aerobic conditions (Figure 3). However, the genes encoding NADP^+^‐specific aldehyde dehydrogenase and β‐ketoadipyl‐CoA thiolase (*boxDE*), which are required to convert 3,4‐dehydroadipyl‐CoA semialdehyde to succinyl‐CoA and acetyl‐CoA (Gescher et al., 2006, Fuchs et al., 2011) were not detected in the *Ca*. D. denitrificans genome. Given these results, *Ca*. D. denitrificans presumably degrades benzoate partially under aerobic conditions to produce formate, which could be utilized for further metabolism such as formate oxidation. Accordingly, *Ca*. D. denitrificans seems to use aromatic compounds derived from secondary metabolites and cell lysates of other ABC members. Indeed, aromatic compounds such as naringenin and flavanone, which are synthesized from L‐phenylalanine, were detected in the centrifugal supernatant of wet biomass scraped from the non‐woven fabric in the anammox bioreactor by metabolome analysis using LC‐TOFMS (Figure A2). However, since the genes responsible for each metabolic step have not been identified although the synthetic pathways of both compounds are known, the producers of such aromatic compounds could not be specified. In general, because the metabolites are usually utilized immediately in the active cell growth phase, these detected aromatic compounds may be the final metabolites that are no longer metabolized or slowly metabolized. Unlike these aromatic compounds, benzoyl‐CoA which is a key intermediate in the anaerobic degradation of many aromatic compounds was not detected and in fact, expression of the genes responsible for benzoyl‐CoA metabolism was observed (Figures 2 and 3). Although it is difficult to specify the origin of aromatic compounds, it is thought that such compounds are presumably supplied to *Ca*. D. denitrificans through the lysis of active ABC members sensitive to the lytic enzymes in addition to autolyzed old cells because *Ca*. N. proteolyticus possesses various lytic enzymes (Okubo et al., 2021).

**TABLE 1:** Module completion ratios of carbon metabolism pathways

Carbon metabolism	Module ID	MCR (%)	
		Genome	TRP
Carbohydrate metabolism			
Glycolysis (Embden‐Meyerhof pathway), glucose => pyruvate	M00001	90.0	30.0
Entner‐Doudoroff pathway, glucose−6P => glyceraldehyde‐3P + pyruvate	M00008	50.0	0.0
Pentose phosphate pathway	M00004	100.0	42.9
Citrate cycle (TCA cycle)	M00009	87.5	62.5
Glyoxylate cycle	M00012	100.0	80.0
PRPP biosynthesis	M00005	100.0	100.0
Aromatics degradation			
Benzoyl‐CoA degradation, benzoyl‐CoA => 3‐hydroxypimeloyl‐CoA	M00541	100.0	100.0
CO_2_ fixation			
Reductive pentose phosphate cycle (Calvin cycle)	M00165	90.9	81.8
Reductive citrate cycle	M00173	90.0	80.0
Reductive acetyl‐CoA pathway	M00377	28.6	0.0
3‐Hydroxypropionate bi‐cycle	M00376	30.8	7.7
Dicarboxylate‐hydroxybutyrate cycle	M00374	46.2	38.5
Hydroxypropionate‐hydroxybutylate cycle	M00375	14.3	7.1

Abbreviations: MCR, module completion ratio; TRP, transcriptome.

As mentioned above, because aromatic compounds are known to inhibit anammox activity and decrease the population of anammox bacteria (Peng et al., 2018; Pereira et al., 2014), the degradation of aromatic compounds by *Ca*. D. denitrificans is expected to help maintain the activity and population of anammox bacteria in the reactor. Interestingly anammox‐enhanced benzene degradation by denitrifiers has been reported, although the enhancement mechanism is not fully understood (Peng et al., 2017). This interrelationship through aromatic compounds seems to be one of the reasons why *Ca*. D. denitrificans and *Ca*. B. pituitae are linked in the anammox bioreactor and may be one reason that makes isolating ABC members difficult. Based on genomic analyses, it is expected that *Ca*. D. denitrificans can degrade aromatic compounds under both aerobic and anaerobic conditions. Since dissolved oxygen in the synthetic medium is consumed by nitrite oxidizer, *Ca*. N. proteolyticus, grown on the surface of biomass, it is thought that an oxic‐anoxic interface occurs between the surface and inside of the biomass with a thickness of several millimeters formed on the non‐woven fabric often used as a carrier of up‐flow anammox bioreactor. This phenomenon was also observed in another type of anammox reactor (Nielsen et al., 2005). Therefore, the presence of both anaerobic and aerobic pathways for aromatic compound degradation would facilitate the colonization of *Ca*. D. denitrificans in the ABC.

*Ca*. D. denitrificans has two formate dehydrogenases, classified as formate dehydrogenase‐N and NAD‐dependent formate dehydrogenase. The genes encoding formate dehydrogenase‐N were organized to encode the α‐ (DSYM_14690), β‐ (DSYM_14700), and γ‐ subunits (DSYM_14710) and the accessory protein FdhE (DSYM_14720). The genes encoding molybdopterin molybdenum transferase (*moeA*; DSYM_14660), molybdenum cofactor guanylyltransferase (*mobA*; DSYM_14670) and GTP 3’,8‐cyclase (*moaA*; DSYM_14680) responsible for biosynthesis of a molybdenum cofactor were adjacent to the genes for formate dehydrogenase‐N. The formate dehydrogenase accessory protein gene (*fdhD*; DSYM_17580) was distant from this gene cluster. The genes encoding α‐ and β‐subunits were expressed with RPKM ratios of 0.4 and 0.3, respectively. In *E*. *coli*, formate dehydrogenase‐N couples formate oxidation to nitrate reduction (Maia et al., 2015), but the expression of the *narGHJI* genes was not detected in our analyses (Figure 1). Thus, formate oxidation by formate dehydrogenase‐N is not necessarily coupled to the reduction of nitrate in *Ca*. D. denitrificans. On the other hand, the NAD‐dependent formate dehydrogenase is considered to couple formate oxidation to the reduction of NAD^+^, providing reducing equivalents in the form of NADH (Maia et al., 2015). The genes encoding this enzyme were organized in the order γ‐, β‐, α‐ and δ‐subunits (DSYM_25570‐600), but the gene encoding the accessory protein (*fdsC*) was missing. In addition, only the α‐subunit gene was expressed with an RPKM ratio of 0.1.

### 3.3 Glucose metabolism

**FIGURE 4:**
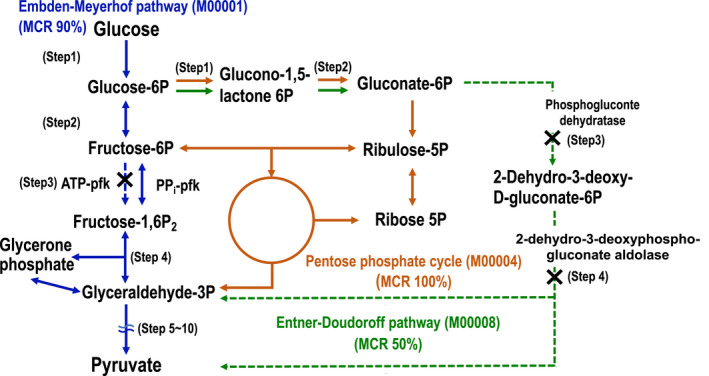
Carbohydrate metabolism pathway. The dotted arrow represents the missing enzyme. Blue, brown, and green lines show the flow of Embden‐Meyerhof (M00001), pentose phosphate (M0004), and Entner‐Doudoroff (M00008) pathways, respectively. Dashed lines show missing reaction steps. Blue and green lines show reversible and irreversible reactions respectively. The brown line shows the reactions focused on in this study

*Ca*. D. denitrificans has 9 of the 10 enzymes of glycolysis (Embden‐Meyerhof pathway; M00001; ie., MCR: 90%; Table S3: https://doi.org/10.5281/zenodo.5089211), but lacks ATP‐dependent phosphofructokinase (ATP‐PFK) (Figure 4). ATP‐PFK is a regulatory enzyme in glycolysis that catalyzes the irreversible phosphorylation of fructose 6‐phosphate to fructose 1,6‐bisphosphate using ATP. *Ca*. D. denitrificans possessed a diphosphate‐dependent phosphofructokinase, which catalyzes the reversible phosphorylation of fructose 6‐phosphate to fructose 1,6‐bisphosphate (PPi‐PFK, DSYM_10110) (Alves et al., 2001; Mertens, 1991). Considering these results, *Ca*. D. denitrificans would have the metabolic potential to route glucose through the Embden‐Meyerhof pathway utilizing PPi‐PFK instead of ATP‐PFK. The Entner‐Doudoroff pathway (M00008) is not functional because *Ca*. D. denitrificans lacks the enzymes phosphogluconate dehydratase and 2‐dehydro‐3‐deoxyphosphogluconate aldolase, which catalyze the last two steps of this 4‐step pathway (i.e., MCR: 50%; Table 1 and Figure 4). On the other hand, since *Ca*. D. denitrificans possessed all enzymes of the pentose phosphate pathway, it can presumably metabolize glucose through this pathway as well as the PPi‐PFK dependent Embden‐Meyerhof pathway. However, since MCRs of these glycolytic pathway modules based on the transcriptome data were low (30.0% and 42.9%, respectively; Table 1), *Ca*. D. denitrificans is not expected to use glucose as a major carbon and energy source in the anammox bioreactor under regular operating conditions.

### 3.4 Inorganic compound metabolism

**FIGURE A3:**
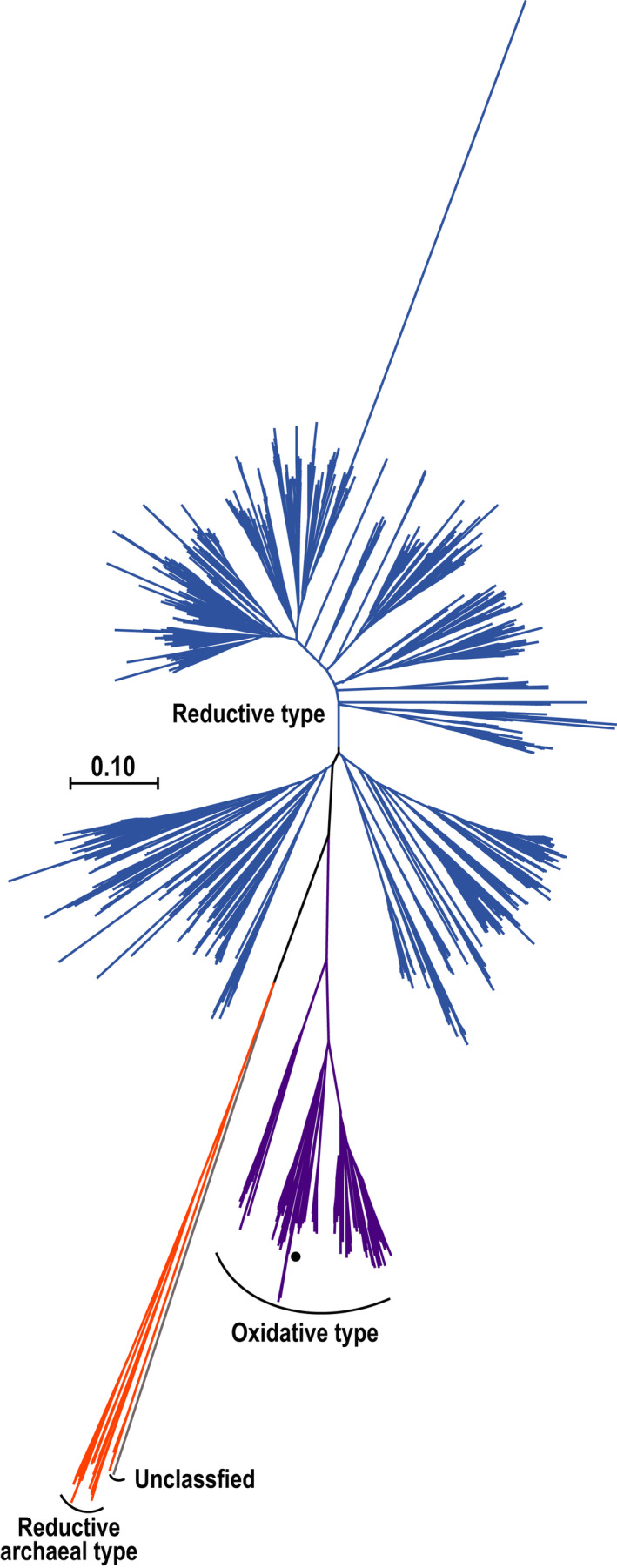
Phylogenetic tree based on concatenated amino acid sequences of DsrAB. The dot represents the phylogenetic placement of the *Ca*. D. denitrificans enzyme

Because inorganic compounds can act as electron donors for denitrification (Capua et al., 2017), we search for genes that would suggest potential inorganic electron donors that could drive denitrification. *Ca*. D. denitrificans possesses two sox gene clusters, *soxXAYZ* (DSYM_07930‐60) and *soxYZAXB* (DSYM_23290‐330) (Table 2), but *soxCD* was not found. The reaction catalyzed by the Sox system in the absence of SoxCD proteins is considered to convert thiosulfate to elemental sulfur (S0) or polysulfide, which produces 2 mol of electrons per mol of thiosulfate (Friedrich et al., 2005). *Ca*. D. denitrificans also possesses *cysDN* (DSYM_2680‐90) and sat (DSYM_23250), *fccAB* (DSYM_09470‐80), *dsrAB* (DSYM_11230‐40) and *aprAB* (DSYM_23230‐40), which are also involved in sulfur metabolism (Table 2). The dissimilatory sulfite reductase (DsrAB) catalyzes the reduction of sulfite to sulfide but DsrAB from sulfur‐oxidizing bacteria catalyzes an oxidative reaction. Since the oxidative and reductive type enzymes are phylogenetically distinct (Müller et al., 2015), a phylogenetic analysis was performed to determine its type. Because the *dsrAB* genes from *Ca*. D. denitrificans clustered with oxidative type enzymes (Figure A3), *Ca*. D. denitrificans is predicted to oxidize sulfide into sulfate via elemental sulfur (S0), sulfite, and adenosine 5′‐phosphosulfate (Kappler & Dahl, 2001; Russ et al., 2014). The oxidation of sulfur compounds can be coupled with denitrification (Chung et al., 2014; Russ et al., 2014) and these reactions can also be catalyzed by enzymes encoded by *cysDN* and *sat*, *fccAB*, and *aprAB*, respectively (Table 2). Thus, *Ca*. D. denitrificans may also contribute to the removal of sulfide, which is known to inhibit anammox activity (Russ et al., 2014).

**TABLE 2:** Gene repertoire and the expression profile for sulfur metabolism

Sulfur metabolism	Enzyme	Gene	Locus ID	RPKM ratio
Thiosulfate oxidation (S_2_O_3_^2−^ → S^0^ or polysulfide)	L‐cysteine S‐thiosulfotransferase	*soxX*	DSYM_07930	0.0
	L‐cysteine S‐thiosulfotransferase	*soxA*	DSYM_07940	0.0
	Sulfur‐oxidizing protein	*soxY*	DSYM_07950	0.0
	Sulfur‐oxidizing protein	*soxZ*	DSYM_07960	0.0
	Sulfur‐oxidizing protein	*soxY*	DSYM_23290	0.0
	Sulfur‐oxidizing protein	*soxZ*	DSYM_23300	0.0
	L‐cysteine S‐thiosulfotransferase	*soxA*	DSYM_23310	0.2
	L‐cysteine S‐thiosulfotransferase	*soxX*	DSYM_23320	0.0
	S‐sulfosulfanyl‐L‐cysteine sulfohydrolase	*soxB*	DSYM_23330	0.2
Sulfide dehydrogenase (H_2_S/HS^−^ → S^0^)	NADPH‐dependent 2,4‐dienoyl‐CoA reductase, sulfur reductase	*fccB*	DSYM_09470	1.1
	Cytochrome *c*‐type	*fccA*	DSYM_09480	0.0
Reverse dissimilatory sulfite reductase (S^0^ → SO_3_^2−^)	Dissimilatory sulfite reductase β subunit	*dsrB*	DSYM_11230	0.0
	Dissimilatory sulfite reductase α subunit	*dsrA*	DSYM_11240	0.0
Adenylylsulfate reductase (SO_3_^2−^ → adenosine 5′‐phosphosulfate)	Adenylylsulfate reductase, subunit A	*aprA*	DSYM_23230	0.4
	Adenylylsulfate reductase, subunit B	*aprB*	DSYM_23240	0.3
Sulfate adenylyltransferase (adenosine 5′‐phosphosulfate → SO_4_^2−^)	Sulfate adenylyltransferase subunit 2	*cysD*	DSYM_26280	0.0
	Sulfate adenylyltransferase subunit 1	*cysN*	DSYM_26290	0.0
	Sulfate adenylyltransferase	*sat*	DSYM_23250	0.0

*Ca*. D. denitrificans possessed four hydrogenase genes (DSYM_11670, DSYM_11790, DSYM_19030 and DSYM_28330). DSYM_11670 and DSYM_28330 were classified into [NiFe]‐hydrogenase Groups 1c and 1e, respectively (Søndergaard et al., 2016). Enzymes of these groups are considered to be oxygen‐sensitive and support anaerobic hydrogenotrophic respiration linked to the reduction of various electron acceptors. However, only DSYM_11670 was expressed with an RPKM ratio of 0.2. The remaining two genes, DSYM_11790 and DSYM_19030, were classified [NiFe]‐hydrogenase Groups 3d and 2b, both of which are O_2_‐tolerant; expression of these genes, however, was not observed.

### 3.5 CO_2_ fixation

**FIGURE 5:**
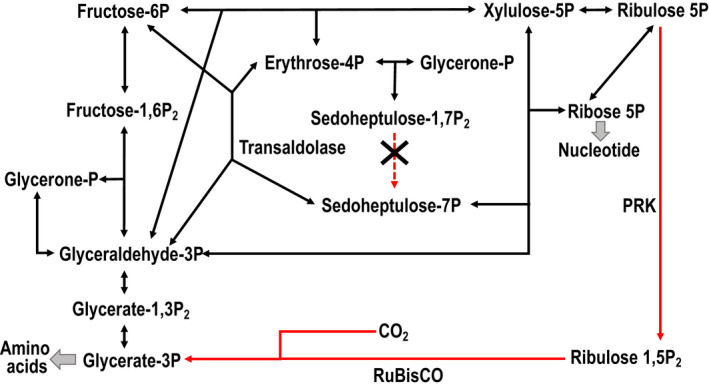
Variant reductive pentose phosphate cycle for CO_2_ fixation pathway with transaldolase substituting for the missing sedoheptulose‐bisphosphatase (missing enzyme represented by the dotted arrow). Red arrows show irreversible reactions. PRK, phosphoribulokinase; RubisCO, ribulose‐bisphosphate carboxylase/oxygenase

**FIGURE A4:**
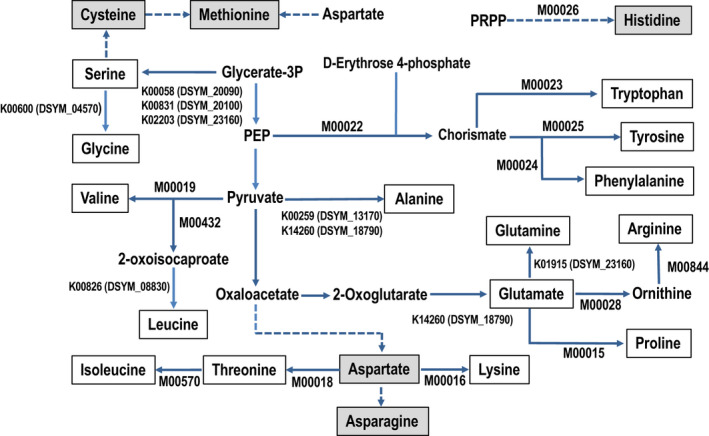
Amino acid biosynthesis pathways. Dotted lines represent missing or incomplete steps. Biosynthetic pathways for shaded amino acids are missing. PEP, phosphoenolpyruvate; PRPP, 5‐Phospho‐alpha‐D‐ribose 1‐diphosphate. K and M numbers represent KEGG Orthology and module IDs

Genome analyses revealed that *Ca*. D. denitrificans has the potential to utilize various inorganic electron donors such as reduced sulfur compounds and hydrogen in addition to organics to carry electrons to the electron acceptor nitrate. Autotrophic denitrifiers fix inorganic carbon (CO_2_ and HCO_3_^−^), but *Ca*. D. denitrificans does not have complete modules for any of the six known carbon fixation pathways (Table 1). *Ca*. D. denitrificans lacks ATP citrate lyase and citryl‐CoA synthetase, which are key enzymes for the reductive citrate cycle, although the MCR of this cycle is high (90%; Table S2: https://doi.org/10.5281/zenodo.5089211). Also, because *Ca*. D. denitrificans lacks the gene encoding sedoheptulose‐bisphosphatase, which catalyzes the 9th reaction step in the 11‐step reductive pentose phosphate cycle (Calvin cycle), the completion ratio of this module was 90.9% (Table 1). This missing enzyme is considered to be unique to the Calvin cycle (Atomi, 2002). On the other hand, it was recently shown that transaldolase (EC 2.2.1.2) can substitute for sedoheptulose‐bisphosphatase and sedoheptulose‐1,7‐bisphosphate aldolase. *Ca*. D. denitrificans possesses a transaldolase (DSYM_09660) gene, although expression of the gene was not observed (Frolov et al., 2019). Therefore, *Ca*. D. denitrificans presumably has the potential to carry out CO_2_ fixation via this irregular Calvin cycle using transaldolase instead of sedoheptulose‐bisphosphatase. The fixed carbon seems to be used for the biosynthesis of amino acids (Figure A4), nucleotides, and sugars (Table 1). Expression of the genes for nine of the ten steps of the irregular Calvin cycle was observed, including genes encoding ribulose‐1,5‐bisphosphate carboxylase/oxygenase (RuBisCO) (DSYM_24900) and phosphoribulokinase (DSYM_24930) (Figure 5). Considering the limited number of metatranscriptomic reads that mapped to the coding regions of *Ca*. D. denitrificans (2937 reads in total), gene expression may be underestimated. Therefore, the Calvin cycle is presumed to work in the anammox bioreactor, even if only at low levels. Nevertheless, since experimental evidence for CO_2_ fixation through the irregular Calvin cycle has been confirmed only in *Thermodesulfobium acidiphilum*, a member of the Firmicutes (Frolov et al., 2019), further biochemical experiments are required to conclude whether *Ca*. D. denitrificans can fix CO_2_ in the ABC. This type of metabolic versatility, which suggests a mixotrophic lifestyle, would be helpful for *Ca*. D. denitrificans to colonize an anammox bioreactor and also natural environments.

### 3.6 Phylogenetic position of *Ca*. D. denitrificans

**FIGURE A5:**
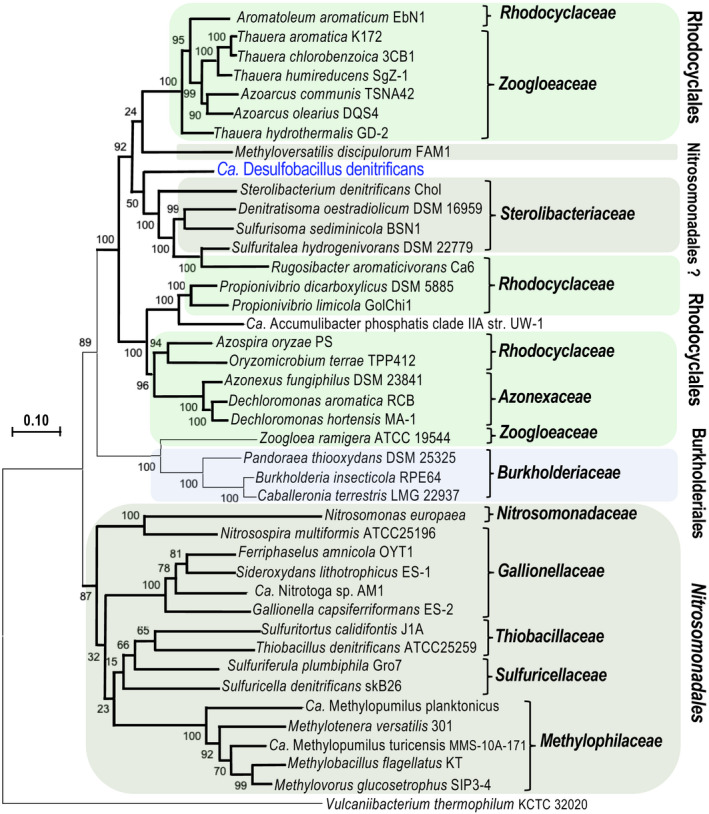
Phylogenetic tree based on concatenated amino acid sequences of ribosomal proteins. Numbers at the nodes are the percentages of 1000 bootstrap replications. *Ca*. D. denitrificans appears in blue character

A phylogenetic tree based on a concatenated alignment of 45 ribosomal proteins was constructed to infer the phylogenetic position of *Ca*. D. denitrificans. *Ca*. D. denitrificans mapped to a large cluster mainly comprised of various species within the order *Rhodocyclales*. However, *Ca*. D. denitrificans formed a cluster with a sub‐cluster comprised of 4 species within the order *Nitrosomonadales* and *Rugosibacter aromaticivorans* within the *Rhodocyclales*, whereas other species within the *Nitrosomonadales* independently formed their large own cluster (Figure A5). Also, *Methyloversatilis discipulotum* within the *Nitromonadales* formed a cluster with a sub‐cluster of *Rhodocyclales*. In short, these five *Sterolibacteriaceae* species within the *Nitromonadale*s were nested in the large cluster of *Rhodocyclales*. Thus, it appears that these species assigned as *Sterolibacteriaceae* species in the *Rhodocyclales* cluster are misidentified and should be reclassified as members of the order *Rhodocyclales*. Although *Ca*. D. denitrificans is seemingly close to species in the *Sterolibacteriaceae* cluster, *Ca*. D. denitrificans is phylogenetically distant from *Sterolibacteriaceae* species due to the low bootstrap value of 50% (Figure A5). Therefore, *Ca*. D. denitrificans is presumed to be a member of a new family within the order *Rhodocyclales*.

## 4 CONCLUSION

Through a series of analyses, it was found that *Ca*. D. denitrificans has versatile potential to exploit various compounds such as aromatic compounds, glucose, sulfur compounds, and hydrogen as electron donors for denitrification, but the most favorable compounds were aromatics, which inhibit anammox. In addition, *Ca*. D. denitrificans also possessed hydroxylamine oxidoreductase, which catalyzes the oxidation of hydroxylamine to NO and potentially the reverse reaction, and the potential for CO_2_ fixation via an irregular Calvin cycle, implying mixotrophic potential. Thus, we revealed the metabolic versatility that may facilitate the colonization of *Ca*. D. denitrificans in the anammox bioreactor. Our findings will not only boost our understanding of the functional relationships between incomplete denitrifiers and anammox bacteria, but also a potential isolation strategy for non‐isolatable anammox community members.

## CONFLICT OF INTEREST

None declared.

## AUTHOR CONTRIBUTIONS

**Takashi Okubo:** Data curation (lead); Formal analysis (equal); Methodology (equal); Visualization (lead); Writing‐original draft (lead). **Hideto Takami:** Conceptualization (lead); Formal analysis (equal); Funding acquisition (lead); Methodology (equal); Project administration (lead); Writing‐review & editing (lead).

## ETHICS STATEMENT

None required.

## Data Availability

RNA sequence (RNA‐seq) data for the anammox bacterial community and the genome sequence of *Ca*. D. denitrificans are available in the NCBI databases with the accession numbers DRA009157 and AP021857, respectively: https://www.ncbi.nlm.nih.gov/sra/DEA009157, https://www.ncbi.nlm.nih.gov/nuccore/AP021857. Supporting Tables (Tables S1–S3) are available at https://doi.org/10.5281/zenodo.5089211
